# Incidental Detection of PSMA-Avid Thymic Squamous Cell Carcinoma on [^68^Ga]Ga-PSMA-11 PET/CT During Initial Prostate Cancer Staging

**DOI:** 10.3390/diagnostics16213637

**Published:** 2026-11-03

**Authors:** Choon-Young Kim, Shin Young Jeong

**Affiliations:** 1 Department of Nuclear Medicine, Kyungpook National University Hospital, School of Medicine, Kyungpook National University, Daegu 41944, Republic of Korea; omh4ever@gmail.com; 2 Department of Nuclear Medicine, Kyungpook National University Chilgok Hospital, School of Medicine, Kyungpook National University, Daegu 41404, Republic of Korea

**Keywords:** prostate-specific membrane antigen, [^68^Ga]Ga-PSMA-11 PET/CT, thymic squamous cell carcinoma, prostate cancer

## Abstract

A man in his 70s with biopsy-confirmed prostate adenocarcinoma underwent gallium-68-labeled prostate-specific membrane antigen (PSMA)-11 ([^68^Ga]Ga-PSMA-11) positron emission tomography/computed tomography (PET/CT) for initial staging. In addition to intense PSMA uptake in the prostate (maximum standardized uptake value [SUVmax], 15.3), PET/CT incidentally revealed a moderately PSMA-avid anterior mediastinal mass (SUVmax, 6.1). Contrast-enhanced chest CT showed a 6.4 cm heterogeneously enhancing anterior mediastinal mass suspicious for an invasive thymic malignancy, with possible invasion of the adjacent lung and pericardium. Multiple left pleural nodules raised concern for pleural metastases. The patient underwent total thymectomy, lung wedge resection, pericardial excision, and pleural nodule biopsies. Histopathology confirmed moderately differentiated thymic squamous cell carcinoma with lung and pericardial invasion and pleural metastasis (pT2N0M1a, TNM stage IVA; Masaoka–Koga stage IVa). Tumor-cell positivity for p40, CD5, CD117 (c-kit), and GLUT1, together with the histopathological and anatomical findings, supported the diagnosis. The patient subsequently completed four cycles of cisplatin, doxorubicin, and cyclophosphamide chemotherapy, with stable disease in the response assessment, while the concurrent prostate cancer was managed with androgen deprivation therapy. This case illustrates the importance of CT morphology and tissue confirmation when an atypically located PSMA-avid lesion is encountered during prostate cancer staging.

##  

**Figure 1:**
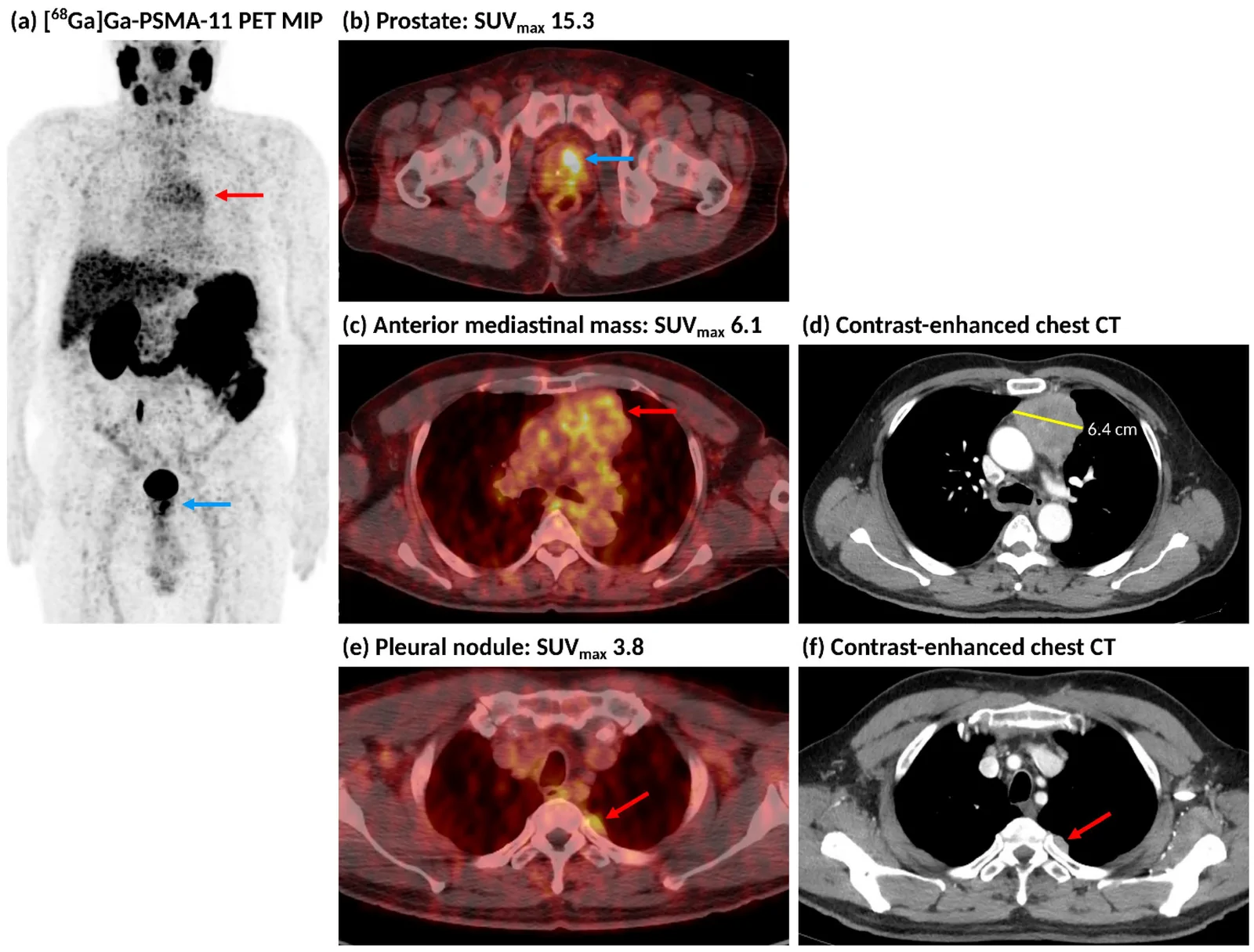
Gallium-68-labeled prostate-specific membrane antigen (PSMA)-11 ([^68^Ga]Ga-PSMA-11) positron emission tomography/computed tomography (PET/CT) and contrast-enhanced chest CT findings in a patient with prostate cancer and incidentally detected thymic carcinoma. A man in his 70s presented with an elevated serum prostate-specific antigen level of 12.3 ng/mL (reference range, 0–4.0 ng/mL). Transrectal ultrasound-guided prostate biopsy revealed adenocarcinoma in all cores obtained from the left lobe (highest Gleason score, 3 + 4 = 7; Grade Group 2; tumor involvement, 50–90% per core). For initial staging, [^68^Ga]Ga-PSMA-11 PET/CT was performed approximately 60 min after intravenous administration of 200 MBq (5.4 mCi) of the tracer. Images were acquired from the skull base to the mid-thigh, with low-dose, unenhanced CT (3.75 mm slice thickness) used for attenuation correction and anatomical correlation. (**a**) Maximum intensity projection (MIP) PET image demonstrates intense PSMA uptake in the prostate gland (blue arrow) and incidental PSMA uptake in the anterior mediastinum (red arrow). (**b**) An axial fused PET/CT image demonstrates intense uptake in the primary prostate lesion (maximum standardized uptake value [SUVmax], 15.3; blue arrow). (**c**) An axial fused PET/CT image demonstrates heterogeneous, moderate PSMA uptake in an anterior mediastinal mass (SUVmax, 6.1; red arrow). (**d**) Contrast-enhanced chest CT reveals a lobulated anterior mediastinal mass measuring 6.4 cm in maximum axial diameter (yellow line), with infiltrative margins. The mass shows heterogeneous enhancement (approximately 50 Hounsfield units [HU] on the low-dose unenhanced CT component of PET/CT and 70 HU on a separate contrast-enhanced chest CT examination) with internal low-attenuation areas suggestive of necrosis. Obliteration of the fat planes between the mass and the adjacent lung and pericardium suggests direct invasion, raising concern for an invasive thymic epithelial malignancy. (**e**) An axial fused PET/CT image at the level of the upper thorax demonstrates mild PSMA uptake in a left pleural nodule (SUVmax, 3.8; red arrow). (**f**) Contrast-enhanced chest CT at the corresponding level shows the left pleural nodule (red arrow). This nodule, the most conspicuous on PET/CT, and several additional smaller pleural nodules along the left hemithorax raised concern for pleural metastases. [^68^Ga]Ga-PSMA-11 PET/CT is widely used for prostate cancer staging because of its high diagnostic performance [1]. However, PSMA uptake is not specific to prostate cancer and may also occur in benign conditions and nonprostatic malignancies [2,3]. As its clinical application expands, recognition and accurate characterization of incidental extraprostatic PSMA-avid lesions have become increasingly important. The differential diagnosis of a PSMA-avid anterior mediastinal mass in this setting included metastatic prostate cancer, thymoma, thymic carcinoma, lymphoma, germ cell tumors, and thymic hyperplasia [4]. Metastatic prostate cancer was considered less likely because PET/CT showed no nodal or bone metastases, and an isolated invasive anterior mediastinal mass would be atypical for metastatic prostate cancer. Thymic hyperplasia was considered less likely given the infiltrative margins and suspected invasion of adjacent structures, and malignant mediastinal germ cell tumors were considered less likely because they predominantly affect younger men aged 20–40 years [4]. However, thymoma, thymic carcinoma, and lymphoma cannot be reliably distinguished on imaging alone [4], and PSMA uptake has been reported in both thymoma and thymic carcinoma [5,6,7]. Therefore, histopathological confirmation was required.

**Figure 2:**
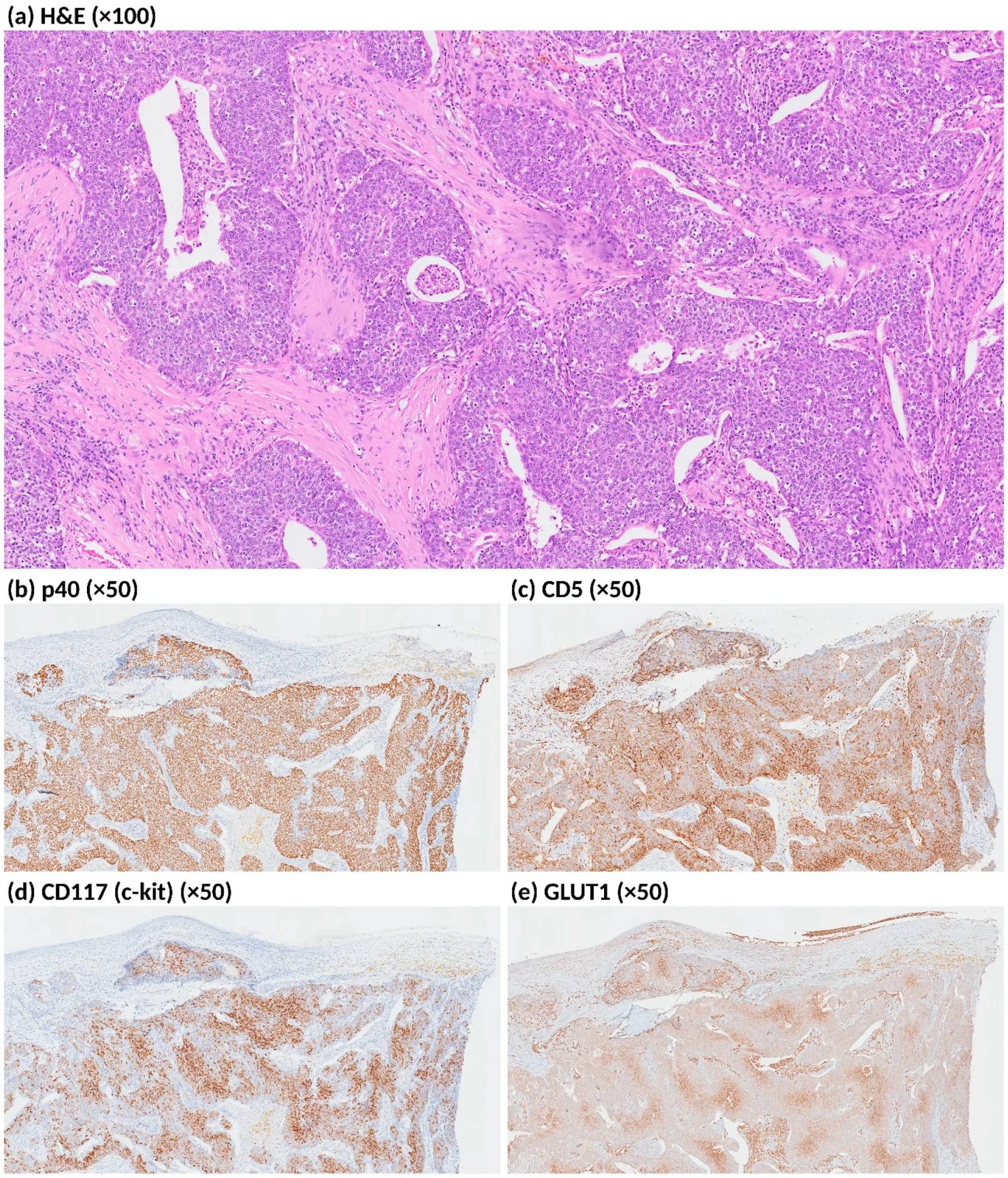
Histopathological and immunohistochemical findings of thymic squamous cell carcinoma. The patient underwent total thymectomy via median sternotomy, accompanied by wedge resection of the left upper lobe, partial pericardial excision, and biopsies of parietal pleural nodules. Histopathological examination revealed an 8.5 × 6.0 cm moderately differentiated thymic squamous cell carcinoma with transcapsular extension and invasion into the perithymic fat, lung parenchyma, and pericardium, without invasion of other adjacent structures. All three resected perithymic lymph nodes were negative for metastasis, whereas one of three resected parietal pleural nodules demonstrated metastatic squamous cell carcinoma. Complete removal of all pleural nodules was not feasible because of their diffuse distribution; therefore, the resection was classified as R2. According to the 9th edition of the TNM classification for thymic epithelial tumors (TETs) [8], the tumor was staged as pT2N0M1a, stage IVA. Direct invasion of the lung and pericardium corresponds to T2, as lung invasion was reclassified from T3 in the 8th edition to T2 in the 9th edition, and the pleural metastasis corresponds to M1a (pleural or pericardial metastasis). This stage corresponds to Masaoka–Koga stage IVa, defined by pleural or pericardial dissemination [9]. (**a**) Hematoxylin and eosin (H&E)-stained section of the resected anterior mediastinal tumor demonstrates an infiltrative tumor composed of nests and sheets of atypical tumor cells separated by fibrous stroma (original magnification, ×100). (**b**) Immunohistochemical staining for p40 shows nuclear positivity in tumor cells, supporting squamous differentiation (×50). (**c**–**e**) Immunohistochemical staining demonstrates tumor-cell positivity for CD5 (**c**), CD117 (c-kit) (**d**), and GLUT1 (**e**) (×50). Co-expression of CD5 and CD117, which is characteristic of thymic carcinoma and uncommon in squamous cell carcinomas of other sites, together with the anterior mediastinal location, supported a thymic origin, and GLUT1 positivity further favored thymic carcinoma over thymoma. These combined findings supported the diagnosis of primary thymic squamous cell carcinoma. Following multidisciplinary discussion among thoracic surgeons, pulmonologists, and urologists, the patient underwent surgical resection. Given the residual pleural disease, systemic chemotherapy was prioritized, and the patient received four cycles of cisplatin, doxorubicin, and cyclophosphamide, an anthracycline-based regimen recommended for advanced TETs in the 2015 European Society for Medical Oncology guidelines [10]. Chemotherapy was initiated approximately 1 month after surgery and completed over 12 weeks, with dose reductions and treatment delays because of poor tolerance. Follow-up CT scans performed approximately 4.5 and 8.5 months after the completion of chemotherapy showed stable disease according to the Response Evaluation Criteria in Solid Tumors (RECIST) version 1.1 [11], with a total follow-up of approximately 12 months after surgery. Further treatment, including local therapy, will be determined according to the subsequent course. Androgen deprivation therapy for prostate adenocarcinoma had been initiated before surgery. Because the stage IVA thymic carcinoma was considered more likely than the localized prostate cancer to determine the patient’s prognosis, treatment of the thymic carcinoma was prioritized, and radical prostatectomy, initially planned after thymic surgery, has been deferred pending the response of the thymic carcinoma. TETs are uncommon neoplasms of the anterior mediastinum, comprising thymomas, thymic carcinomas, and thymic neuroendocrine neoplasms [12]. Contrast-enhanced chest CT plays a central role in evaluating the anatomical extent and invasive features of thymic malignancies, while histopathological examination is essential for definitive diagnosis [13]. Whereas the [^18^F]fluorodeoxyglucose PET/CT features of TETs have been extensively investigated, including their association with histological subtype, stage, and prognosis [14], PSMA uptake in these tumors remains poorly characterized. The published literature includes three case reports describing PSMA uptake in TETs with specified histological subtypes and SUVmax values: mild uptake in a basaloid thymic carcinoma (SUVmax, 3.6) [5], a rare subtype accounting for less than 5% of thymic carcinomas [15], intense uptake in a type B2 thymoma (SUVmax, 24.5) [6], and moderate uptake in a type AB thymoma (SUVmax, 6.0) [7]. An additional PSMA-avid anterior mediastinal tumor has been illustrated in a pictorial review without quantitative or detailed histological data [3]. To our knowledge, PSMA uptake in thymic squamous cell carcinoma, the most common histological subtype accounting for approximately 70% of thymic carcinomas [12,15], has not been specifically documented. Moreover, the uptake of the present tumor (SUVmax, 6.1) was comparable to that of the type AB thymoma and lower than that of the type B2 thymoma. Given the small number of reported cases, the degree of PSMA uptake cannot currently be used to distinguish thymic carcinoma from thymoma, and further studies with more cases are needed to clarify the relationship between PSMA uptake and histological subtype. Furthermore, because PSMA uptake also occurs in benign conditions [2,3], SUVmax alone cannot reliably distinguish benign from malignant extraprostatic lesions; in the present case, malignancy was suggested by the invasive CT features and confirmed histologically. PSMA expression in the tumor-associated neovasculature has been described in various nonprostatic malignancies [16]. However, PSMA immunohistochemistry was not performed in the present case, which is a limitation of this report; therefore, the relative contributions of tumor-cell expression and tumor-associated neovasculature to the observed PSMA uptake remain undetermined. Correlation of PSMA PET findings with PSMA immunohistochemistry in future cases is needed to clarify the basis of PSMA uptake in thymic carcinoma. The present case demonstrates that thymic squamous cell carcinoma may exhibit moderate PSMA uptake and be incidentally detected during initial prostate cancer staging. Although [^68^Ga]Ga-PSMA-11 PET/CT can reveal unexpected extraprostatic malignancies, PSMA uptake alone cannot establish the origin or histological nature of a lesion. Therefore, atypically located PSMA-avid lesions should be carefully evaluated using CT morphology and clinical context, with histopathological confirmation when indicated.

## Data Availability

The data presented in this study are available on request from the corresponding author. The data are not publicly available due to institutional policy and patient privacy restrictions.
